# A Review of Using Mathematical Modeling to Improve Our Understanding of Bacteriophage, Bacteria, and Eukaryotic Interactions

**DOI:** 10.3389/fmicb.2021.724767

**Published:** 2021-09-21

**Authors:** Kathryn M. Styles, Aidan T. Brown, Antonia P. Sagona

**Affiliations:** ^1^School of Life Sciences, University of Warwick, Coventry, United Kingdom; ^2^School of Physics and Astronomy, University of Edinburgh, Edinburgh, United Kingdom

**Keywords:** bacteriophage, mathematical modelling, phage therapy, simulations, stochasticity, heterogeneity, communicable disease, antibiotic alternative

## Abstract

Phage therapy, the therapeutic usage of viruses to treat bacterial infections, has many theoretical benefits in the ‘post antibiotic era.’ Nevertheless, there are currently no approved mainstream phage therapies. One reason for this is a lack of understanding of the complex interactions between bacteriophage, bacteria and eukaryotic hosts. These three-component interactions are complex, with non-linear or synergistic relationships, anatomical barriers and genetic or phenotypic heterogeneity all leading to disparity between performance and efficacy in *in vivo* versus *in vitro* environments. Realistic computer or mathematical models of these complex environments are a potential route to improve the predictive power of *in vitro* studies for the *in vivo* environment, and to streamline lab work. Here, we introduce and review the current status of mathematical modeling and highlight that data on genetic heterogeneity and mutational stochasticity, time delays and population densities could be critical in the development of realistic phage therapy models in the future. With this in mind, we aim to inform and encourage the collaboration and sharing of knowledge and expertise between microbiologists and theoretical modelers, synergising skills and smoothing the road to regulatory approval and widespread use of phage therapy.

## Introduction

### Antimicrobial Resistance

According to the World Health Organization (WHO), only three communicable diseases (lower respiratory infections, diarrheal diseases and tuberculosis) were in the top ten killers globally in 2016, a drop on previous reviews (Ghebreyesus et al., 2018). We are now living over a third longer than 100 years ago (Office for National Statistics, 2017), which is largely attributable to a reduction in infectious diseases through improvements in sanitation and to the widespread use of antibiotics (Ribeiro da Cunha et al., 2019). Antibiotics kill multiple bacterial strains indiscriminately (they are broad spectrum) and therefore allow for treatment with limited pressure on diagnostic sensitivity and specificity. Nevertheless, this broad-spectrum capability is a two-edged sword and can lead to over-prescription, disruption of the gut microbiome, allergic reactions (Lin et al., 2017) and unwanted side effects (e.g., the nephrotoxicity of colistin (Moulin et al., 2016)). However, by far the most significant problem, connected with antibiotic use is antimicrobial resistance (AMR) (O’Neill, 2016).

In spite of their current relatively low global incidence, communicable diseases are certainly not a phenomenon of the past (U.S. Department of Health and Human Services and U.S. Centers for Disease Control and Prevention, 2019). We are now entering the ‘post antibiotic era’ (U.S. Department of Health and Human Services and U.S. Centers for Disease Control and Prevention, 2019), that Fleming warned about in his Nobel prize acceptance speech (Fleming, 1945). It is unlikely that a panacea alternative for antibiotic-resistant infections will arise, and instead novel treatments often have to be approached at a narrower spectrum pathogen-specific level, requiring a greater understanding of individual host–pathogen interactions (HPIs). Possible alternative treatments include novel vaccines, the use of immunotherapy (stimulating or dampening the immune system for a therapeutic effect; Naran et al., 2018) and phage therapy (O’Neill, 2016). Of the options for alternative treatments, the therapeutic use of bacteriophages (phage therapy) is a particularly attractive choice for the treatment of drug-resistant pathogenic bacteria, partially due to their abundance in nature.

### Bacteriophages and Phage Therapy

Phage therapy can be applied without indiscriminate disruptions to the gut microbiota and, because they are hugely abundant and varied in nature, the pipeline for novel bacteriophages is endless. In fact, pathogen-specific bacteriophages have regularly been shown to accumulate specifically alongside their host in its natural habitat, e.g., in hospital effluents and waste water (Lin et al., 2017; Leungtongkam et al., 2020). One great appeal of phage therapy therefore, is the potential to administer lytic bacteriophages as a cocktail or combinatorial therapy, targeting multiple pathogenic molecules and systems, thereby reducing the chance of resistance developing (Smith and Huggins, 1983; Cisek et al., 2017; Abedon, 2019). Bacteriophages also have the ability to co-evolve alongside their bacterial target, creating a ‘self-improving’ treatment (Kysela and Turner, 2007). Phage therapy is also ‘auto-dosing’ and will replicate to match the burden of infection in a specific location (Payne and Jansen, 2000; Schooley and Strathdee, 2020). Importantly, phage therapy has not yet been linked with anaphylaxis or the other side effects associated with some antibiotics in clinical trials (Cai et al., 2017; Gordillo Altamirano and Barr, 2019).

### Barriers to Phage Therapy

The many benefits of phage therapy beg the question of why it is not currently a mainstream treatment option; there are no approved mainstream bacteriophage medicines in the European Union or United States and no phage therapy has yet successfully completed Phase II clinical trials. The barriers to the development of the potentially lifesaving phage therapy into clinical products can be divided loosely into regulatory and experimental factors (although the root cause of regulatory barriers arguably also lies in a lack of fundamental scientific knowledge). Although predating the discovery of antibiotics, bacteriophage research slowed after the advent of antibiotics due to the latter’s broader spectrum and more predictable results *in vivo*. Experimental research on bacteriophages has historically been plagued by unpredicted or unexplained results and a lack of reproducibility between studies (Gordillo Altamirano and Barr, 2019). For example, in a 2017 review covering over 60 anti-*Escherichia coli* O157:H7 phages, there was a significant reduction in *E. coli* titres in the majority of *in vitro* trials, but this was typically not seen in subsequent *in vivo* studies (Sabouri et al., 2017). Unlike the *in vitro* environment, the *in vivo* environment is also affected by the eukaryotic immune system, anatomical barriers, immune status and underlying health conditions, not all of which have been accounted for in *in vitro* studies. Another source of discrepancy between *in vivo* and *in vitro* phage therapy research, is the development of phage resistant isolates (Smith and Huggins, 1983; Cieslewicz and Vimr, 1997; Cairns et al., 2009; Chan et al., 2016; Kortright et al., 2019). This is particularly complex as not only can a bacterium develop resistance to a phage, a phage can also evolve strategies to overcome these resistance mechanisms (Borin et al., 2021). The stochasticity of evolution and co-evolution can make treatment outcomes unpredictable (Sabouri et al., 2017; Abdelkader et al., 2019). In order to progress phage therapy, we need to understand tripartite phage-human-bacteria relationships, rather than just the dipartite human-bacteria interactions that are required to understand treatment with non-replicating agents like antibiotics.

### Overcoming Barriers to Phage Therapy Using Mathematical Modeling

A possible route to better understanding the complex tripartite relationship between phage, bacteria and human, is an increased and routine use of mathematical modeling, combined with experimental lab work. However, biologists are often unfamiliar with mathematical modeling, from the different types of model that are available, to their specific data requirements. The intention of this paper is to provide an introduction to the different types of model, what they can produce, how much and what type of input is required: bridging the gap between experimental biology and theoretical modeling and urging researchers to approach experimental design with an application in mathematical modeling in mind.

### An Introduction to Mathematical Modeling

In theory, modeling is a simplified version of reality (Stopar, 2009). A successful and detailed mathematical model should, in an ideal world, be able to make *a priori* predictions about behavior and the outcomes of future laboratory experiments. Failing this, it should at least inform the selection of experimental variables to be tested, streamline experiments and offer plausible explanations of unexpected results seen *in vitro* and *in vivo*. Although a mathematical model may never be ‘right’ and fully represent the real, complex biological world (McNamara, 2013), this does not prevent it from being useful. Mathematical modeling can be particularly beneficial when there are barriers to experimentation, such as cost, complexity and ethical considerations.

The uses of mathematical models are wide ranging and they have been used to study anything from the interaction dynamics of human immune cells and bacteria during infection (Magombedze et al., 2006; Jayasundara et al., 2019), to collectively modeling individual immune responses in outbreaks (Kucharski et al., 2020) and vaccination programs (Reyes-Silveyra and Mikler, 2016), to finding the most likely causative gene regulation scenario for an observed expression output (Sturm et al., 2011; Dresch et al., 2013; Bowyer et al., 2017) or reconstructing the metabolome of intracellular pathogens (Thiele et al., 2013). The type of model and the input requirements will depend on the research question being asked and the level of detail or depth of information that is required. For example, gaining an understanding of phage pharmacodynamics, requires a detailed model that quantitatively predicts rates of phage and bacterial growth and death (Cairns et al., 2009; Roach et al., 2017). For the understanding of evolution and the development of resistance to phages on the other hand, a schematic, game-theory type model, based on the relative fitness of different bacterial and phage evolutionary strategies may be preferred (Turner and Chao, 1999; Tago and Meyer, 2016). Whereas to characterize entire infectious systems including interactions with the human host, coarse-grained spatial simulations or metabolome models (Islam et al., 2019; Rienksma et al., 2019) would be more useful.

An example of the successful use of a mathematical simulation to study the interaction between antibiotics and a bacterial infection is provided by the tuberculosis model of Aljayyoussi et al. (2017). *Mycobacterium tuberculosis* is an intracellular bacterium, notorious for chronic infections and extensive treatment regimens. Therapeutic outcomes for *M. tuberculosis* are difficult to predict, due to its complex relationship with the eukaryotic host and its phagocytosis into macrophages. In this 2017 study, an imaging-based mouse-model approach was combined with mathematical modeling to make predictions about multiple hypothetical treatment scenarios (Aljayyoussi et al., 2017). Taking into consideration the nutritional constraints of the intracellular environment and the barrier of the host-cell membrane (hindering antibiotics from reaching the target pathogen), the output was a recommendation for treatment with higher doses of the drug rifampicin compared to what was previously used. Independently published in the same year, Boeree et al. (2015, 2017) showed the successful clinical application of these predicted treatment doses and durations in phase II trials (clinical trial NCT01392911). Had collaborations starter earlier, time and resources could hypothetically have been saved. The aim of this paper is to encourage fruitful collaborations between studies like these, adapting methods to the study of phage therapy instead of antibiotics.

### Overview of the Paper

In the section “Types of Model,” we summarize various examples of mathematical model that have been previously used to understand host-pathogen interactions (HPIs), involving bacteriophages where relevant. These examples are loosely grouped into four categories: logical, network, reaction rate and complex (or combined) spatial simulation models (although there are overlaps), but our review is not intended to be exhaustive. In the section “Current Phage Therapy Models” we review current models of phage therapy and highlight key features that have been shown to be important in the creation of realistic biological models of phage therapy, offering suggestions for future modeling endeavors in section “Future Phage Therapy Models.”

General two-component bacterial infection models have unsurprisingly been much more extensive and widespread than models for phage therapy. This review therefore draws many examples from two-component bacteria-eukaryotic host–pathogen interactions (HPIs) and two-component bacteria-phage interactions as well as examining three-component phage-bacteria-eukaryote interactions (two-component viral-eukaryotic HPIs are beyond the scope of this paper). Where phage therapy model examples are not available, we focus in particular on intracellular bacterial infection models, as these share some of the complexities of phage therapy, such as the importance of anatomical barriers.

When discussing two-component host-pathogen interactions, the term ‘host’ will be reserved for the eukaryotic host (e.g., a human patient or mouse model) and ‘pathogen’ for the invasive bacterial species. For three-component bacteriophage systems, a pathogen is further defined as the immediate ‘bacterial host,’ whereas the human patient or mouse model is to be termed as the extended ‘eukaryotic host’ for the bacteriophage (and for the bacterial pathogen) (Keen and Dantas, 2018). The terms ‘two-component’ or ‘three-component’ are used to define the number of distinct biological entities or ‘agents’ involved in the system being modeled, i.e., anything with replicative ability, but does not exclude the possibility of subpopulations arising through genetic heterogeneity, differentiation, or the presence of non-biological components (e.g., antibiotics).

## Types of Model

### Logical Models

**FIGURE 1:**
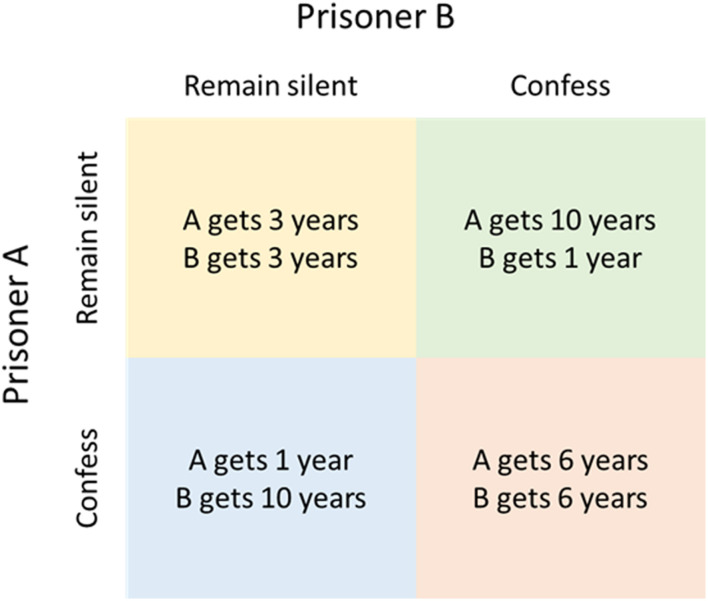
Outcome matrix for the prisoner’s dilemma.

The simplest models that can be used to examine biological relationships are logic-based analyses of biological ‘strategies’ using game theory. Classical game-theory models are often used by social scientists and are based on rational decision making and ‘pay offs’ from choices made by the various ‘players’. Examples include the famous ‘prisoner’s dilemma,’ in which the two players are prisoners who must choose whether to ‘defect,’ i.e., confess, in order to obtain a shorter sentence for themselves, at the expense of a longer sentence for the other player, or ‘cooperate’ with each other by remaining silent, resulting in an intermediate sentence for both (Figure 1). This leads to three possible outcomes: either both confess (defect), which results in a long sentence for both, both remain silent (co-operate) or one remains silent and the other confesses, each with a fixed pay-off for each player in the different outcome scenarios (Figure 1). The crucial feature is that the payoff for each player’s actions depends upon the actions of the other player, which can lead to paradoxical results. In the prisoner’s dilemma, the optimum strategy for both players taken individually is always to defect, no matter what the other player does, even though this results in a worse outcome for each player than mutual co-operation.

In evolutionary game theory (EGT), this conflict between individual advantage and collective benefit is applied to situations where the players are different organisms and the payoff is evolutionary fitness (Nowak and Sigmund, 1999), i.e., the probability of survival or the opportunity to reproduce. For these reasons, EGTs can be particularly useful when assessing evolutionary strategies, important in trying to understand resistance developing to phage therapy. Much as in the prisoner’s dilemma, EGTs can be used to explain escalating levels of aggressive and costly host-pathogen interactions (mutual defection), as opposed to more favorable commensal or symbiotic interactions (mutual co-operation). An example of a simple EGT, with inputs and outputs close to the prisoner’s dilemma, was produced by Nowak and Sigmund (1999); Turner and Chao (1999). Here, the two ‘players’ were the populations of a phage and its mutant, which had a different infection rate or multiplicity of infection (MOI). The strategies of co-operating and defecting were represented by the manufacturing and sequestering of diffusible shared products, respectively. The result of this were an evolutionary drive toward defecting or ‘selfishness,’ even though this came at a cost compared to co-operating. Like the prisoner’s dilemma, this result is paradoxical as a population were both are defectors has a lower fitness than one containing only co-operators.

While this study exactly paralleled the simple prisoner’s dilemma, there are many more potential complexities to be taken into account in biological interactions. Even in Turner et al’s paper, the outcome was in reality influenced by the relative size of each phage population, which was not a feature of their simple model. An example of a more complex EGT, where the players are of different species, was provided by Tago and Meyer (2016); Table 1, where the transition of obligate intracellular pathogen *Ehrlichia ruminantium* from a virulent to an attenuated form via *in vitro* passaging in cell culture versus the *in vivo* environment, was characterized in a repeat host-pathogen game. The ‘players’ in this scenario were the intracellular bacterium and the eukaryotic host. There were four possible strategies, two for each player. The eukaryotic host had strategies of incomplete defense, i.e., the intracellular immune system only, represented in the *in vitro* environment and complete defense, represented *in vivo*, with intra- and extracellular components of the immune system present. The pathogen on the other hand had the strategies of ‘evasion before replication’ (coming with the cost of evading the immune system) and ‘replication without evasion’ (i.e., concentrating all resources on replicating; Table 1). The game-theoretic model developed showed that attenuation would result from the trade-off between the benefit to the bacterium of evasion of the immune system *in vivo* (leading to increased virulence) and the cost of maintaining this evasive genotype *in vitro*, where the immune system is absent. In an *in vivo* scenario, the eukaryotic host had a dominant strategy, i.e., a fully functional immune system would always predominate, but the pathogen did not have a dominant strategy (i.e., a strategy with the best pay-off versus costs). The pay-off and cost to the pathogen of becoming attenuated entirely depended on the status of the host immune system and changed over time, dependent on the number of passages the players underwent. This scenario therefore required the inclusion of repeated sequential games, where timescales were factored in, and the pathogen did not have a dominant strategy until a ‘second round,’ when the host had adopted a strategy.

**TABLE 1:** Outcome matrix for the study by Tago and Meyer (2016), where L is the gain of the bacterium if it succeeds to replicate, *s* is the probability of success, *c* the energy cost of evading the host’s defense system and *r* is replication.

	*In vitro* (incomplete defense: intracellular)	*In vivo* (complete defense: intra- and extracellular)
Evasion before replication	L - c, -k(L - c)	sL - c, -k(sL - c)
Replication without evasion	rL, -krL	0,0

More complex game theory models have also been used to predict antibiotic resistance profiles (Chowdhury et al., 2019) and in the same way, could be used to understand phage-bacteria co-evolution and the chances of non-lytic commensal relationships versus resistant populations developing (Borin et al., 2021). These more complex games may have more than two strategies and result in more than three possible outcomes or more than one state of equilibrium (Venkateswaran and Gokhale, 2019). For example, outcomes are also influenced by the frequency of ‘events,’ i.e., contact events due to population densities and the number of replication cycles, meaning strategies can change over time as pay-offs alter. The games are also not pairwise and there are multiple individuals within a population, each undergoing replication cycles at different rates and capable of each producing their own mutations and distinct survival mechanisms. Finally, unlike the traditional prisoner’s dilemma, ‘eliminated populations’ also play a role in EGTs and although a ‘selfish’ strategy may come with a fitness cost or a decrease in virulence, the cost is outweighed by the alternative outcome of death (Smith and Huggins, 1983; Chan et al., 2016).

Despite these added complexities, the input requirements for an EGT model can be very simple and will often just be a qualitative list of possible strategies together with assumptions about how these interact within specific timescales. For example the variables presented by Tago and Meyer (2016) are the relative costs and benefits of various strategies to either agent, plus the relative probabilities that particular strategies will succeed (Table 1). The input to this type of model is not necessarily based directly on quantitative data and can often be qualitative, meaning the outcome of predictions is also often qualitative, with output just revealing which strategy will be dominant or ‘more likely’ when a stable equilibrium is reached. For example, it may reveal whether it is more beneficial for a pathogen to prioritize antibiotic resistance versus immune system evasion and how this will alter under different biological scenarios (e.g., during antibiotic treatment). Pay-off values may simply be −1 for a negative impact on fitness or +1 for a positive impact, for example. For this reason, these game-theoretic models do not always require any additional wet lab experiments or quantitative parameters. However, wet lab experiment can be used to provide the assumptions, probabilities or outcomes, and associated timescales required to build an EGT model.

### Network Models

**FIGURE 2:**
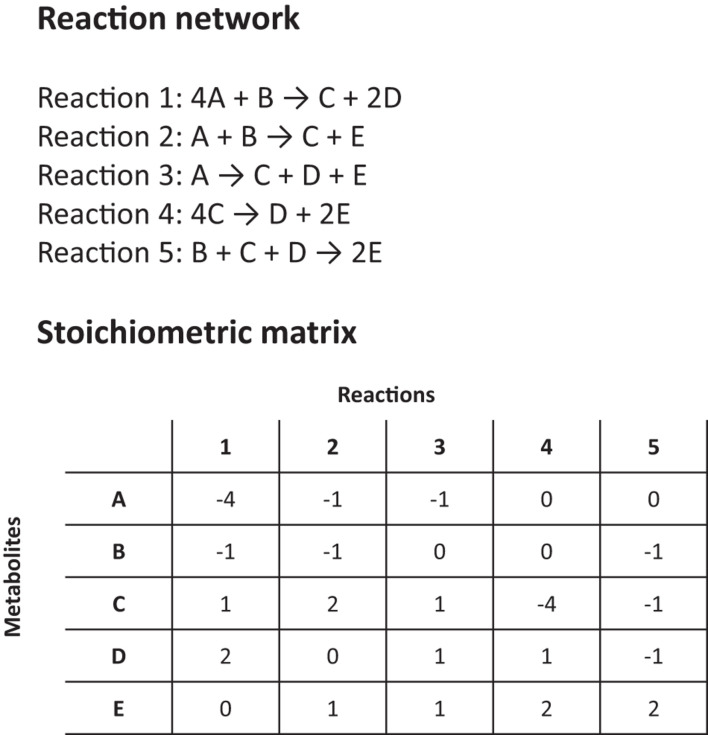
Details on a simplified reaction network and stoichiometric matrix for a flux balance analysis (FBA).

Another major tool for modeling HPIs is flux-balance analysis (FBA). FBA estimates the rates of metabolite production and consumption within a cell by using existing genetic and reaction-network information and making simplifying assumptions to render the problem mathematically tractable. It can be likened to a constrained flow chart of cellular reactions, where the impact of gene knockouts, perturbations and drug inhibition on fitness and metabolite production can be studied (Raman and Chandra, 2009; Putra and Lyrawati, 2020). For a ‘whole cell’ or metabolome model, an FBA is surprisingly simple (Figure 2). No extensive kinetic data is required for a basic FBA model and all the necessary information can be obtained ‘in one go’ by annotating a gene sequence and using biochemical knowledge to predict the possible reaction pathways or enzyme functions (i.e., based on homologies with similar enzymes). Then, using the known exponential growth rate of an organism to scale the relative stoichiometries of each reaction, the amount reaction contributes to a phenotype can be calculated (Figure 2; Orth et al., 2010). Given certain key assumptions, which we discuss below, FBA can then output the rates of individual reactions, or how general quantities, like the growth rate of the organism, vary under different environmental conditions, e.g., after the administration of a therapeutic drug or as a result of genetic changes (Orth et al., 2010; Rowe et al., 2018).

The simplicity of FBA models is achieved by the use of purely ‘relative’ values for reaction rates and by making two key assumptions. The first, steady-state assumption, is that the reactions have reached equilibrium so that the concentrations of all products and reactants are conserved (this is what ‘flux-balance’ refers to). The second assumption is that the organism is evolutionarily optimized in one or more ways, e.g., to achieve a maximum overall growth rate or the maximum production of some bio-product. Combining these two assumptions permits the mathematical technique of ‘linear programming’ to be applied, which enables the rapid calculation of all reaction rates across networks consisting of thousands of genome-wide pathways, without the need for high-powered computers and without the need for individual reaction rates to be known *a priori*.

There are, of course, some limitations to FBAs. Firstly, there are knowledge gaps in all genomic-scale reconstructions and we do not know the role of all genes (Orth et al., 2010), FBAs cannot be used to predict metabolic concentrations (only flux at steady state) and do not necessarily account for regulatory effects, e.g., that certain genes and their associated metabolic pathways will often be switched off. The predicted outcome from an FBA will therefore be incorrect if, for example, a key metabolic pathway has not been included. However, this is a high-throughput modeling technique that offers the ability to assess the effect of a wide range of initial conditions, e.g., gene knock outs or the removal of substrates or enzymes, on the rerouting of metabolic networks and bottlenecks. As such, FBAs are extensively used in drug discovery (Shen et al., 2010; Krueger et al., 2016) as well as biosynthesis, e.g., to map out the metabolic networks of microbes used in fermentation (Wang et al., 2017), in order to help save money and time in wet lab experiments. Similarly, FBAs could be used to study the possible perturbations to the flux of metabolites within a pathogenic host when resources are diverted to phage production (Islam et al., 2019). This may be particularly relevant when trying to understand the role of lysogenic versus lytic lifecycles adopted by phages.

Adding further constraints to a basic FBA model can make it more realistic, e.g., assigning directionality or reversibility to a reaction, imposing maximum or minimum reaction rates (where those are known) (Orth et al., 2010), adding details of gene regulation, considering nutrient availability in different anatomical locations or the rate of metabolite absorption through anatomical barriers (Rienksma et al., 2018). Adding such realistic constraints could help explain discrepancies between *in vitro* and *in vivo* phage therapy testing (Sabouri et al., 2017). Recently, increasingly complex two-component FBA models have been created, which map the metabolomes of both a eukaryotic host and pathogen simultaneously, revealing the reciprocal effects on the metabolism of each agent. An FBA model of this type was published by Rienksma et al. (2019) to study *M. tuberculosis* metabolic responses to hypothetical drug regimens. This model (sMtb-RECON) combined previously developed maps of the *M. tuberculosis* (Jamshidi and Palsson, 2007; Rienksma et al., 2014) and human metabolism (Thiele et al., 2013; Swainston et al., 2016) and included 8987 reactions and 6373 metabolites. However, this model also included extra information from paired transcriptomes, produced in parallel from the host and pathogen, as well as information on phagosome-specific cytosolic nutrient availability, metabolite uptake and secretion rates. The result was a more realistic and detailed FBA model than previously seen for a disease scenario with *M. tuberculosis*. Another example of a multi-organism FBA model was produced by Islam et al. (2019) for the bovine rumen bacterial gut microbiome. In this 2019 study, the functional role of the rumen virome on three key bacterial gut organisms was investigated, looking at how the metabolic functions of bacteriophages associated with their bacterial hosts, studying metabolic exchange and the re-programming of bacterial carbon metabolism when in the presence of phages and one another. Following on from this, three-component phage therapy FBAs (including both the bacterial and eukaryotic host) are not out of the question, where uptake and resource availability implications associated with phage replication can be considered in the context of both the immediate bacterial and extended eukaryotic host. Thoroughly annotated genomic data already exists on the human host as well as a range of pathogens including *E. coli* (Orth et al., 2010; Zeng and Yang, 2019), *Staphylococcus aureus* (Lee et al., 2009; Shen et al., 2010) and *Pseudomonas aeruginosa* (Raman and Chandra, 2009), creating a ‘parts catalogue’ (Kauffman et al., 2003). Hypothetically therefore, just additional data on a phage of interest would need to be collected in order to study the metabolic dependencies in these tripartite interactions. This is not a trivial extension however and is limited by the need for fully annotated genome sequences for bacteriophages. The role of many bacteriophage genes and how precisely they are regulated, is not yet known however, even for well-studied phages like T4 and T7 (Dunn et al., 1983; Miller et al., 2003). Additional work will therefore be required to elucidate the role of different bacteriophage (and some bacterial) genes and regulatory elements and how these may interfere with nutrient resources and the regulation of normal gene expression in their hosts. In order to make the models truly representative of a clinical scenario, the role of spatial variables in the interactions of phages with anatomical and bacterial barriers will also need to be considered (Rienksma et al., 2019). Over time, the databases available on different metabolomes will evolve, making the options for three-component FBAs easier and more realistic, with the potential of even creating dynamic kinetic mechanistic models of combined metabolomes.

### Reaction Rate Models

**FIGURE 3:**
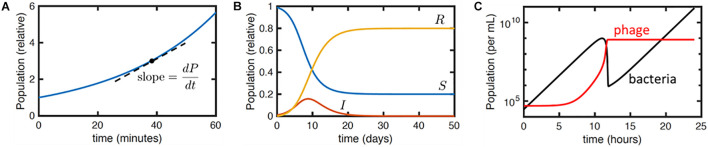
Illustration of differential equation models. **(A)** Plot of equation Y for exponential growth *P* = *P*_0_*e*^*at*^ with *P*_0_ = 1 and *a* = 1.7 hr^– 1^. Dashed line indicates the meaning of the derivative*dP*/*dt*. Here, *dP*/*dt* = *aP*. **(B)** Numerical solution to the SIR model (Equations 3a–c) with initial conditions *S*(0) = 0.99,*I*(0) = 0.01, *R*(0) = 0 and parameter values *k* = 1 days^– 1^ and *r* = 0.5 days^– 1^. The qualitative behavior is that the infection *I* grows exponentially and then dies away as the density of susceptible hosts *S* reduces, leaving a fraction *R* of the population resistant. **(C)** Numerical solution to Equations 4a–d, with typical parameter values taken from Cairns et al. (2009): *a* = 1 h^– 1^, *f* = 10^– 5^ h^– 1^, *b* = 10^– 7^ mL/h, *h* = 1.5, t = 10 min, and starting susceptible and viral concentrations *S*(*t* = 0) = 3 × 10^4^ cfu/mL and *P*(*t* = 0) = 5 × 10^4^ pfu/mL, respectively, with no infected or resistant cells. The plot shows the total bacterial (black) and viral (red) concentrations over time.


Therapeutically, bacteriophages do not follow linear kinetics like other pharmaceuticals. They are self-replicating and ‘auto-dosing’ (the rate of phage growth is dependent on the host population or degree of infection) and so the study of pharmacokinetics (what the body does to a drug) cannot be separated from the study of pharmacodynamics (what the drug does to the body). It is important therefore to understand the non-linear relationship between bacteriophage and bacteria in an *in vivo* environment in order to make optimal use of phage therapy and avoid unpredicted results. For this, a reaction-rate model can be very useful. Ordinary differential equations (ODEs) are widely used to model interactions that involve rates or changes over time (Ewald et al., 2020). In detail, an ODE model describes the mathematical relationships in concentrations or populations of any of a number of species, e.g., cells, viruses, nutrients or toxins, and the rates at which such quantities change (Ewald et al., 2020). For example, ODEs can be used to model the exponential growth rate of bacteria based on data input on the population size (*P*) at any one time (*t*) and the corresponding growth rate (*a*) (Equations 1 and 2; Figure 3). During exponential growth, the more bacteria there are, the quicker the population grows, and the number of new bacteria produced in an hour will depend on the population size at the beginning of that hour (i.e., the rate of change in the population over time is equivalent to the growth rate multiplied by the population). Mathematically, the equation is the following:

(1) $$\frac{d\,P}{d\,t}=a\,P,$$

which has solution


(2) $$P=P_{0}\,e^{a\,t}.$$

Here *dP/dt* means the derivative of *P* with respect to *t.* It is the slope of the *P* vs. *t* curve and here it is the instantaneous bacterial growth rate. *P*_0_ is the initial population size and *e* = 2.718 (Euler’s number, a constant and the base of the natural logarithm).

A more complex set of coupled differential equations could be used to express interactions between organisms or between bacteria and antibiotics, e.g., the famous compartmental susceptible-infected-removed (SIR) model (Ross, 1916; Equations 3a–c) for the time course of an infectious disease


(3a) $$\frac{d\,S}{d\,t}=-k\,I\,S,$$

(3b) $$\frac{d\,I}{d\,t}=k\,I\,S-r\,I,$$

(3c) $$\frac{d\,R}{d\,t}=r\,I,$$

where *S* is the population size of some susceptible host organism or cells, *I* is the infected host population and *R* represents the removed population, where ‘removed’ can imply either dead or resistant cells or organisms. The parameters *k* and *r* are the rates of infection and death (or resistance development), respectively. The term *kIS* indicates that infection is due to contacts between infectious and susceptible organisms or cells and that the contact rate is proportional to the product of the two populations, as one would expect by analogy to a basic bimolecular chemical reaction. Unlike logic and network models, this set of equations does not have a simple, analytical solution, but numerical solutions and analytical approximations can readily be derived.

Typically, ODEs are used to predict temporal dynamics. Using the slightly more complex PDEs (partial differential equations; those with more than one independent variable) one can also study spatial effects (Klann and Koeppl, 2012). Both ODEs and PDEs are deterministic (the result is always the same and depends only on the initial conditions) and local in time and space. More realistic extensions include stochastic differential equations (SDEs), which take account of random variation in behavior, e.g., in individual cell division times or drug response; and delay differential equations (DDEs), where memory of past states of the system can be included. These extensions are more costly to implement, and more analytically challenging, but there is often a profound difference between the predictions of stochastic and deterministic models in particular (Renshaw, 1991; discussed more later).

As an example of a rate based model, Cairns et al. (2009) used DDEs to study the development of resistance among *Campylobacter jejuni* populations treated with bacteriophages. The mathematical model developed (simplified slightly by neglecting their phage decay term) was


(4a) $$\frac{d\,S}{d\,t}=a\,S-f\,S-b\,S\,P,$$

(4b) $$\frac{d\,R}{d\,t}=a\,R+f\,S,$$

(4c) $$\frac{d\,I}{d\,t}=-b\,S\,\left(t-\tau \right)\,V\,\left(t-\tau \right)+b\,S\,P,$$

(4d) $$\frac{d\,P}{d\,t}=h\,b\,S\,\left(t-\tau \right)\,V\,\left(t-\tau \right)-b\,S\,P,$$

where *S*, *R*, *I* and *P* are the concentrations of susceptible bacteria, resistant bacteria, infected bacteria and phages, respectively, all evaluated at time *t*. *S*(*t*−τ) and *P*(*t*−τ), etc., refer to these concentrations evaluated at time *t*−τ, where τ is the latent period. The other parameters are: *a* the growth rate of susceptible and resistant bacteria (to account for a trade-off between phage resistance and fitness a lower growth rate could instead be specified for the resistant bacteria); *f* the rate at which the resistance mutation arises per susceptible bacterium; *b* the rate constant for phage binding to susceptible bacteria; and *h* the phage burst size. This is a DDE because it involves quantities evaluated at earlier times, i.e., *S*(*t*−τ) and *P*(*t*−τ).

This model gives typical curves as shown in Figure 3C. The initial peak in bacterial concentration corresponds to the growth and then extinction of the phage-susceptible bacterial population, while the resistant population grows exponentially at long times. The phage population grows initially but then plateaus as the concentration of susceptible bacteria falls. Fitting these curves to relatively simple experimental data, specifically the concentration of bacteria and phage obtained over time, together with known parameters such as the phage burst size, enables estimates of the other phage and bacterial physiological parameters specified in the previous paragraph. This permits the estimation of further derived parameters, like the threshold in bacterial concentration required for phage replication, which are relevant to phage-based treatments.

Multiple differential equations can also be combined and incorporated into larger models to simulate more complex scenarios. An example of a complex system of coupled ODEs is the modeling of the dynamics of *Salmonella enterica* infecting macrophages by Gog et al. (2012), where multiple bacterial cells infect a single host cell. This model is an extension of the SIR model, expanded to include separate variables indicating host cells infected by one, versus two, three or more bacteria, with the bacteria themselves being modeled explicitly. By extending the SIR model, it could reproduce more complex data, and allow for more precise fitting of relevant parameters (however, an increased number of free parameters is also dangerous, as it can give a falsely good fit with a poor model: the appropriate statistical techniques must be applied rigorously to compare fits with multiple models). Gog et al. created sixteen candidate models to include or exclude all combinations of four features (intra-cellular bacterial replication; death of infected cells; re-infection rate differing from the rate of the first infection and the presence of one versus two populations of host cells (i.e., differentiated macrophages, each with different susceptibilities to infection)). Experimental information input was quantitative and included basic infection rate, the effects of multiplicity of infection (MOI) on infection rate, growth rate of bacteria and the death rate of infected macrophages, with the theoretical model being used to fit to the number of bacterial cells infecting a macrophage over time. Often, the best fit will come from the model with the largest number of degrees of freedom, but in this work, a statistical test (a maximum likelihood estimation (MLE)) showed that including just two of the four features (different re-infection rate and differentiation of macrophages) explained the data sufficiently well, with no significant advantage provided by the other two features. Another example of a reaction rate model is the work of Wood et al. (2014) who used a similar model to study intracellular *Francisella tularensis* infections, identifying the role of heterogeneity in susceptibility to infection of individual hosts within a population. The methods utilized in these studies could be adjusted to study phage infection of bacteria, as opposed to bacterial infection of human cells, looking at the effect of re-infection and superinfection (where a pre-existing viral infection prevents a secondary infection).

We have here discussed differential equations only at a cell or population level: simple differential equations can also be used to understand sub-cellular molecular details (Scott et al., 2010; Mayorga et al., 2018), but where cellular compartments, or spatial processes such as diffusion matter, a more complex combined model may be more appropriate (Krone, 2004).

### Combined Complex Modeling Techniques

Aspects of the previously discussed modeling techniques (plus many more besides) can be coupled together in a single complex spatiotemporal simulation, as in, e.g., Cowan et al. (2012). These complex models are produced by combining ‘layers’ of information, e.g., communication networks, resource ‘transactions,’ population densities, access to nutrients (Schreinemachers and Berger, 2011) and are based on the idea that a small number of common processes underlie a wide variety of cellular functions, e.g., forces of attraction in protein-protein binding (O’Sullivan and Perry, 2013). Historically, ‘spatial simulation models’ have been used to characterize ecological systems (Wallentin, 2017) or in epidemiological studies (Karl et al., 2014) but this could also be applied microscopically to phage-bacteria-eukaryote relationships at a cellular level (Cowan et al., 2012).

Hypothetically, we should be able to model an entire cell at a molecular level (Klann and Koeppl, 2012), developing a ‘virtual lab’ or ‘virtual cells’ (Cowan et al., 2012). Due to the current technological limitations on the processing of simulations, however, molecular models are often limited to smaller systems or fast processes. For this reason, these models are often ‘coarse grained’ (Levitt, 2014; Kmiecik et al., 2016), i.e., they are simplified, in order to study larger systems and timescales. For example, ‘pseudo-molecules’ made of coarse grained ‘beads’ (Ingólfsson et al., 2014; Casalini, 2020) containing simplified representations of amino key side chains may be used to represent complex proteins versus using detailed atomic information from an x-ray crystallography derived protein structure (Levitt and Warshel, 1975); population dynamics models may be coarse grained to the level of individual cells or cellular populations, as we saw in the section “Reaction Rate Models.” Despite this current limitation, however, this kind of model can have high predictive power and produce usable quantitative values for future experimentation. Fine grained data is not always necessary for the desired result. For example, coarse-grained spatial simulation models have been used to study the formation of phage plaques in soft agar (Krone, 2009) and how environmental spatial structure and biofilms impact on phage-bacteria interactions (Bull et al., 2018).

**FIGURE 4:**
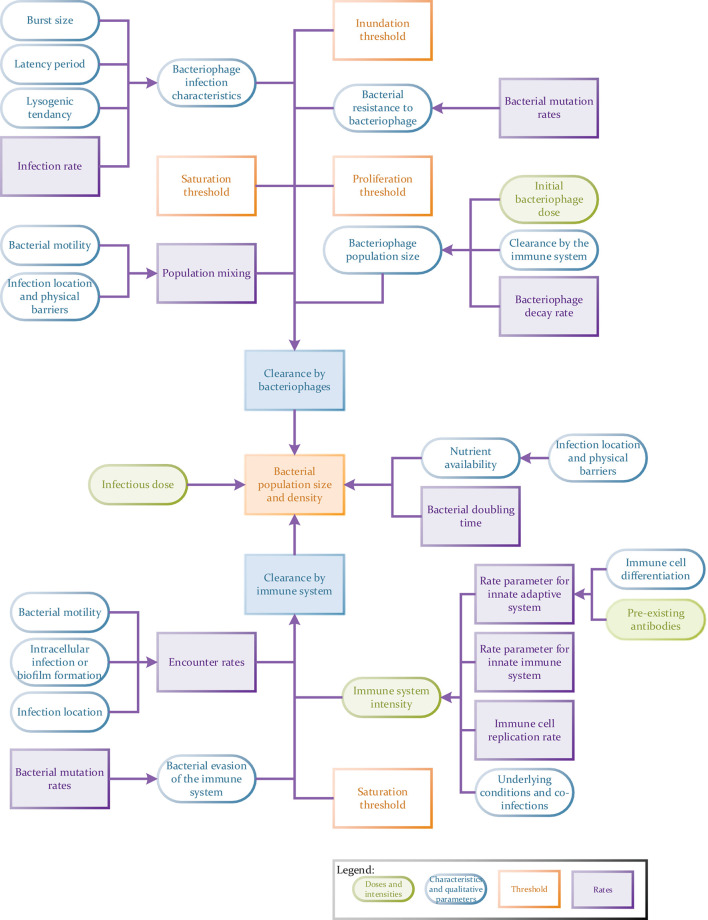
Flow chart to illustrate the potential complexity of a tripartite phage therapy model taking full account of host/pathogen/phage interactions.

Nevertheless, combined modeling approaches are much more demanding in their requirement for quantitative data than the models previously discussed here, e.g., EGT models. In general, the more parameters one can measure independently for a full spatial simulation model, the better (Figure 4). These models may require, but are not limited to, the input of robust data on population densities, treatment agent diffusion, clearance and mixing rates (Klann and Koeppl, 2012), types of host and pathogen populations (i.e., proportions of treatment-resistant versus susceptible bacteria or specific host immune cells versus non-specific cells; Wood et al., 2014), infection location (e.g., intracellular infections) and the associated nutrient availability in this location (Aljayyoussi et al., 2017), the presence of pre-existing antibodies (Majewska et al., 2015), host immune status (Lathrop et al., 2018) as well as the presence of any co-infections (competition between pathogens). Other variables may also include host (bacterial and eukaryotic) infection rates, bacterial doubling times, rates of bacterial transmission across anatomical barriers (Cowan et al., 2012), host-pathogen population mixing rates (as a result of anatomical barriers, pathogen motility and the formation of biofilms or aggregates) (Wikle and Royle, 2002), rate of pathogenic clearance by the innate or adaptive immune system, the rate of development of treatment resistance or immune system evasion methods. Data on all of these variables can be analyzed using multiple sub-models. For example, phage resistance could be examined using a combination of EGTs and ODEs. The chances of a model being used to make successful predictions will also be dependent on how thorough data collection is (i.e., the number of relevant interlinking dependents and variables factored in) and how representative it is of a real-world situation. For example, *in vitro* host infection rates do not necessarily carry forward to *in vivo* studies where anatomical barriers also play a role in controlling infection (Lathrop et al., 2018) and the access a drug has to a pathogen.

## Current Phage Therapy Models

Modeling a system with three biological entities is significantly more complicated than modeling a two-component HPI or a model with a eukaryotic host, bacteria and an inanimate antibacterial treatment, due to the fact that a bacteriophage is self-replicating and can evolve genetically (it is a ‘self-improving’ treatment) (Payne and Jansen, 2000). For this reason, many existing phage models are two-component phage-bacteria models rather than three-component models (also involving the eukaryotic host). However, experimental findings are highlighting, more and more, the complex relationship between not only a bacteriophage and bacterium, but also between the bacteriophage and the eukaryotic host. For example, bacteriophages have been shown to play a significant role in the gut microbiome (where they make up an estimated 90% of the gut population; (Wu and Ross, 2016; Górski et al., 2018; Keen and Dantas, 2018)), and there is an indication of cross-talk between phages and the innate immune system (Tiwari et al., 2011; Lin et al., 2017; Roach et al., 2017) as well as a phage role in immune homeostasis, although these are not fully understood. The bacteriophage-bacterial-human relationship is thus a fully interconnected tripartite relationship, rather than a sequential three-component relationship where the intermediate bacterium just connects the phage and eukaryote. With the complexity of these relationships, the use of mathematical modeling has been and could be extremely beneficial in assisting experimental lab work and, we believe, of great importance in the future of phage therapies. We now give examples of how current models of phage therapy have been utilized, what they have shown and what some of the limitations and knowledge gaps are, with the idea of inspiring the development and use of these kinds of models in future studies. These examples have been loosely divided into models based on phage relationships with their immediate bacterial host and the innate or adaptive arms of the extended eukaryotic host. Many of these models used rate- based differential equations, sometimes in combination with logical models. To date, large FBAs and spatial simulations with three-components have not been produced. Following from this analysis of previous research, we recommend areas for future study and highlight key features to produce more accurate and robust mathematical models in the future.

### Relationship Between Bacteriophages and the Bacterial Host

In the non-linear relationship between bacteria and bacteriophages, the replication potential of a bacteriophage is largely dependent on the properties and status of its immediate host, meaning that bacteriophage replication is reliant on the entity they are consequently removing (Payne and Jansen, 2000; Cairns et al., 2009; Roach et al., 2017). Models created by Payne and Jansen (2000) and Cairns et al. (2009) underlined two important threshold parameters that need to be considered in mathematical models of these self-replicating pharmaceuticals: the minimum concentration of bacteria needed for phage replication (proliferation threshold) and the minimum concentration of phages for a bacterial population to decline (inundation threshold) (Payne and Jansen, 2000; Cairns et al., 2009). The inclusion of these important parameters is essential in understanding therapeutic phage dosages. Other mathematical models have also highlighted the need to include the feature of bacterial resistance to phages and resulting resultant outgrowth in simulation studies (Cairns et al., 2009; Leung and Weitz, 2017; Roach et al., 2017).

### Relationship Between Bacteriophages and the Eukaryotic Host Innate Immune System

Previous failures in reproducing results from *in vitro* studies in *in vivo* phage therapy scenarios can be partially ascribed to an incomplete understanding of the interactions between bacteriophages and the innate immune system (Leung and Weitz, 2017). In fact, multiple studies have shown that the immune system needs to be functional for phage therapy to be successful and that the two systems work synergistically (Tiwari et al., 2011; Górski et al., 2017; Roach et al., 2017; Van Belleghem et al., 2018). Immunocompromised or neutropenic subjects (i.e., with a low neutrophil count) have not responded as well as subjects with fully functional immune systems to phage therapy (Tiwari et al., 2011; Roach et al., 2017), and two-component *in vitro* studies (with no immune cells) often result in a lysogenic co-existence between phages and bacteria (Leung and Weitz, 2017). In the absence of human cells therefore, a valid treatment may appear to be less efficacious than it would be in a clinical environment. It is not until the immune system is added to experimental and computational phage therapy models that the equilibrium shifts toward bacterial removal (Van Belleghem et al., 2018). In particular, the presence of neutrophils is critical to the success of phage therapies (Tiwari et al., 2011; Roach et al., 2017). Reaction rate models created by Roach et al. (2017) estimated that the presence of 20–50% fully functional neutrophils is required for a phage therapy to be successful (Roach et al., 2017).

Against a high infectious load, phage therapy cannot succeed without the immune system (Tiwari et al., 2011; Leung and Weitz, 2017; Roach et al., 2017). The immune system can reach saturation (i.e., there is a finite number of killer cells produced in a given time and there is a maximum rate at which they can destroy invaders (Leung and Weitz, 2017)), bacteria can develop ways of evading the immune system (e.g., biofilm formation (Olson et al., 2002)) and phage cannot replicate when bacteria are below a threshold level (Payne and Jansen, 2000). Eradication of the bacterial host will result in termination of phage replication and the immune system is required to ‘mop up’ these remaining bacteria. This ‘immunophage’ synergy mirrors the action of some bacteriostatic antibiotics, which are also dependent on the immune system to clear infections once the pathogen has stopped replicating (Gordillo Altamirano and Barr, 2019). Including this partnership between the innate immune system and phages will likely be crucial in producing realistic predictions and outcomes from *in silico* phage therapy models.

### Relationships Between Bacteriophages and the Eukaryotic Host Adaptive Immune System

There is currently less information on any synergy between phages and the eukaryotic adaptive immune system than on the innate immune system (Leung and Weitz, 2017), but there has been some evidence of the clearance of bacteriophages by the adaptive immune system (Merril et al., 1996; Hodyra-Stefaniak et al., 2015; Majewska et al., 2015; Van Belleghem et al., 2018) and IgG-type antibodies have been shown to be active against bacteriophages (Hodyra-Stefaniak et al., 2015; Majewska et al., 2015). Although the presence of anti-phage antibodies has not always prevented a favorable outcome from phage therapy (Van Belleghem et al., 2018), the reason for this is little understood. It is therefore clear that a greater understanding of the mammalian host-versus-phage (MHvP) immune response is also key to understanding phage therapies and producing successful models. For example, the outcome of the production of anti-phage antibodies has been shown to be influenced by the therapeutic phage dosage, route of administration, application schedule and varies on a phage-to-phage basis (Majewska et al., 2015). More long term experiments may also be needed to reveal the implications of the immune system for the development of antibodies against phages (as well as the long term changes in bacterial resistance to phages) (Kortright et al., 2019).

### Conclusion on Existing Three-Component Models

It is clear that a number of factors have been key to creating realistic mathematical models of phage therapy, including the synergy between the immune system and bacteriophages (Tiwari et al., 2011; Górski et al., 2017; Roach et al., 2017; Van Belleghem et al., 2018), the mammalian host-versus-phage (MHvP) immune response (Merril et al., 1996; Hodyra-Stefaniak et al., 2015; Majewska et al., 2015; Van Belleghem et al., 2018), threshold densities of phages and bacteria (Payne and Jansen, 2000; Cairns et al., 2009) and the development of phage resistant bacterial populations (Cairns et al., 2009; Leung and Weitz, 2017; Roach et al., 2017). However, there are a number of knowledge gaps and limitations with current two- and three-component mathematical models that also still need to be addressed in order for phage therapy models to accurately predict experimental outcomes *a priori*, particularly in a clinical scenario. For example, it is important to perform long term *in vivo* studies to better understand if there is a synergistic role of the adaptive immune system in phage therapy. In addition, there are limitations relating to the omission of the natural stochasticity present in nature from models, e.g., variations in antibody numbers from person to person, possibly due to the intentional simplification of models. Having discussed some of the different types of models available, we now look to put this into the context of future research and how others may apply modeling to their own work.

## Future Phage Therapy Models

Here we discuss considerations for the choice of a mathematical model and the depth of information required before presenting the importance of introducing stochasticity into models. Following on from this, we make observations on key features that should be addressed in future models and how this can be done. There are an extensive number of qualitative and quantitative parameters and features which *could* be included in models (Figure 4). These include initial concentrations of phage and bacteria, concentration thresholds, rates and population ratios, to name a few. However, we highlight a specific few priorities, selected due to a relative lack of inclusion in a number of previous models (see the section “Current Phage Therapy Models”) or because other previous models have shown that inclusion of these variables have considerably improved mathematical model accuracy (Guttman et al., 2005; Cairns et al., 2009; Leung and Weitz, 2017; Roach et al., 2017). The features we prioritize are genetic stochasticity and heterogenous populations, as well as the temporal dynamics of biological time delays, relating to the spatial dynamics of population densities and mixing.

### Choosing an Appropriate Model

#### Resolution of Data Required

In a laboratory setting, a less complicated *in vitro* approach may be more appropriate for an in-depth study of a microscopic parameter, such as cell division or infection rates, whereas animal models are required to study overall effects on pathogenesis. *In vitro* macrophage-cell-line experiments and similar, offer an insight into the role of the innate immune system in controlling pathogenic infection, whereas whole organism *in vivo* studies provide an insight into both innate and adaptive arms of the eukaryotic immune system. In the same way, different types of mathematical model also need to be selected depending on the research question. Modeling approaches vary in the complexity of their input and output: from the input of quantitative versus qualitative data to outputs ranging from precise numerical predictions to purely qualitative descriptions of possible outcomes (Table 2). When designing models, it is important to find a balance between models being too simplistic compared to overly complex (Stopar, 2009), i.e., to produce a realistic model without the modeling or experimental burden becoming problematic.

**TABLE 2:** Summary of approaches to modeling host pathogen interactions.

Model type	Data input demand	Temporal modeling	Spatial modeling	Computational cost	Stochastic
Logical/game theory	Very Low	No	No	Very low	No
Network/FBA	Low	Yes	Yes	Low	No
Differential/reaction rate	Intermediate	Yes	No	Intermediate	Sometimes
Combined complex models	High	Yes	Yes	High	Sometimes

Although models are arguably more realistic when a greater depth of data is included, this complexity will not always be required and a more ‘coarse grained’ or simplified model can often be used. An analogy for this would be that in the study of traffic routes, we do not need to include data on the color or brand of cars, this is unnecessary information which will likely overcomplicate the model (O’Sullivan and Perry, 2013). As a biological example, a population may be simplified and represented as two distinct ‘wild type’ and ‘resistant’ populations, where all mutants are combined in one group rather than accounting for all the multiple heterogenous mutant populations that may have appeared, each with different mutations and fitness costs (Cairns et al., 2009). In the same way, when studying phage receptor binding, biomolecules may be coarse grained into key functional groups rather than atoms (Merchant and Madura, 2011; Casalini, 2020).

Breadth of information is also something which needs to be considered when planning modeling and experiments. For example, if one wanted to understand pathogenic bacterial fitness in different nutrient environments, then sequencing of the genome and the production of a single organism FBA may tell a scientist everything they need to know (Lee et al., 2009; Rowe et al., 2018; Zeng and Yang, 2019). In order to understand this in a clinical setting however, the production of paired transcriptomes of the pathogen and eukaryotic host and their use in a more complex two-component FBA would be more informative, providing information on how infection dynamics and pathogen fitness depend on the status of the eukaryotic host (Islam et al., 2019; Rienksma et al., 2019). The study of regular antibiotics can also be added to this system and predictions made on the changes in flux of metabolites depending on this treatment agent (Shen et al., 2010; Krueger et al., 2016). To extend this to study the effects of a phage therapy however, a third organism would need to have its genome sequenced and a three-component FBA produced. None of these FBA models are ‘wrong,’ but each will answer a different question and will have its own place in biology.

If collecting reproducible data from multiple different experimental approaches produces a more robust data set, it is important that modeling is also performed from multiple angles. Modeling based on logic models should be used to complement and corroborate predictions from network and reaction rate models and vice versa. As an extension of this, *in vitro*, *in vivo* and *in silico* data should also be cross-compared for consistency to allow for the synergistic expansion of our understanding of models and their realism (Ewald et al., 2020).

Closely linked to deciding on the depth and breadth of knowledge required for a model and the choice to simplify features, is the choice between approaching a model from a stochastic or deterministic point of view, a topic which will now be discussed.

#### Stochastic Versus Deterministic Models

Spatial simulation models and to some extent rate-based models, can be described as having either a deterministic or stochastic approach. A deterministic model, making links between outcomes and causative events, will always produce the same output from a given starting condition or state, as there is no variability. For example, a model may make the assumption of a rigid genetic causation of a particular hereditary trait. A stochastic model on the other hand has a random probability distribution and so an outcome cannot be predicted precisely, since there will be statistical variation. Stochastic variations may therefore appear as biological ‘noise’ (Wilkinson, 2009) and can relate to diffusion in complex cellular environments (Bressloff, 2014), variations in individual cell division times or the production of heterogeneous cell populations (Wilkinson, 2009), both through differentiation (Gog et al., 2012) and genetic mutation. Although historically deterministic modeling has been very popular (Gill, 2009; Meehan et al., 2020), if one thing has become apparent from previous experimental and modeling research into phage therapy, it is that the biological world is stochastic by nature (Beiting and Roos, 2011). Models therefore have sometimes been too deterministic to fully represent an *in vivo* scenario (Gill, 2009). However, both deterministic and stochastic models do have relevant roles to play in the study of any particular system (Renshaw, 1991). Knowledge gaps in simpler models are often filled with deterministic ‘assumptions’ to make them work, e.g., assuming there is no bacterial replication during a short reaction time (Go et al., 2014). A deterministic approach can be ‘wrong’ or incomplete but is often easier to apply than a stochastic model and can still give helpful insight and may be sufficient. A model does not need to be ‘right’ or complete to be useful (O’Sullivan and Perry, 2013; see also the section “Resolution of Data Required”). (It is also important to note that the relevance of applying a deterministic versus stochastic approach will depend on the population size being analyzed. If the sample size is small then a stochastic model will be necessary (Renshaw, 1991) whereas stochastic effects like mutation can often be treated deterministically in large populations).

In order to specifically study stochasticity, an SDE (stochastic differential equation) may be particularly useful, as are more complex models with multiple possible routes or outcomes. These more complex models often contain calculations know of as ‘branching processes’. This means that the model accounts for biological ‘decisions,’ e.g., a mutation event, where an outcome will alter the path an individual may follow, resulting in a model which looks similar to a family tree. By analyzing two possible outcomes (survival versus extinction; Lashari and Trapman, 2018) at each branch point, this kind of model can, for example, provide information on fluctuations in population size and the differential effects of control mechanisms (e.g., transcriptional regulation) on individuals over time (Athreya, 2006; Meehan et al., 2020) based on the different routes individuals follow (and such information is not accessible to deterministic models). For this reason, branching processes have historically been used to study long term evolution, reproduction and extinction, for example in the study of the spread of epidemics (Lashari and Trapman, 2018; Fyles et al., 2021). An example of a study of stochasticity in infection was produced by Wood et al. (2014), where the heterogeneity in susceptibility to *F. tularensis* infection of individual phagocytic hosts within a population was investigated (Wood et al., 2014). This project used a Markov chain, a stochastic model produced using a chain of rate-based equations to describe a sequence of possible events, in which the probability of each event depends only on the outcome of the previous event. In the context of the study by Wood et al. (2014) there were three possible bacterial ‘events’: birth (cell division or release of bacteria from phagocytes), death and survival (phagocytosis without cell death). The output of this model was information on the relationship between the infectious dose of the pathogen and eukaryotic response (i.e., the onset of symptoms).

As can be seen, the study of stochasticity in mutation and the development of genetic heterogeneity within a population is very relevant to a number of studies (Cairns et al., 2009; Gog et al., 2012; Wood et al., 2014). We now discuss the relevance of genetic heterogeneity in bacteriophage research and highlight it as a priority for future modeling scenarios.

### Priority Areas for Future Research

#### Genetic Heterogeneity

The implications of genetic heterogeneity and the resulting resistance to bacteriophages in making accurate predictions from models was highlighted in the work of Cairns et al. (2009) studying the treatment of *C. jejuni* infections. They found that the inclusion of data on the development of phage-resistant bacteria was critical to producing a model matching real world outcomes. In addition to bacterial evolution, bacteriophages can also co-evolve and develop their own strategies to combat bacterial resistance (Borin et al., 2021). Every stage in the bacterial pathogen and phage life cycles is susceptible to mutations that will alter the balance in the phage–host relationship, can result in heterogenetic sub-populations (Edwards et al., 2016) and will come with potential fitness costs or survival benefits. For example, changes to a bacterial genome can result in the development of resistance to a treatment, evasion of the immune system or changes to growth and infection rates (e.g., through prioritizing replication over virulence) (Stopar, 2009). The ratios of each of these sub-populations will vary over time (García et al., 2019) and will have reciprocal knock-on effects on each of the other agents, resulting in different ‘strategies’ and outcomes in battles for ‘survival of the fittest’ (Turner and Chao, 1999; Cairns et al., 2009; Tago and Meyer, 2016). It is important therefore to consider the rate of, and probability of, evolutionary mutations and population heterogeneity in future models, to increase their realism (Diard et al., 2017). To do this, one needs to understand the stochasticity of mutations, e.g., through the study of phage receptors, bacterial resistance mechanisms and phage counter adaptations, including the approximate rates at which they develop. This data could help to explain some unexpected wet lab data (Sabouri et al., 2017) and suggest new ways to prevent or slow down the development of phage resistance among pathogenic bacteria. Bacterial resistance to antibiotics is well known and avoiding this same path with alternative treatments will be critical to our future relationship with infectious diseases (Fleming, 1945).

Game theory models, particularly those with multiple ‘rounds,’ could be particularly useful to identify the key strategies that are most likely to dominate evolutionarily and would bring the greatest ‘reward’ for agents. Due to the stochasticity associated with genetic heterogeneity, models with multiple ‘decision points’ (branching processes) and stochastic differential equations (SDEs) would also be helpful, studying processes in the context of a ‘memory’ of previous events (Gog et al., 2012; Wood et al., 2014; see previous section). For example, branching processes have already been used to better understand the development of AMR (Meehan et al., 2020). Modeling data also needs to be analyzed in the context of changes to ‘payoffs’ and probabilities over time (Tago and Meyer, 2016), depending on the resistance phenotype developed. For this kind of analysis, a delayed differential equations (DDEs) may be very useful. As well as developing our understanding of phage-bacterial host evolution and co-evolution, collecting more information on how the eukaryotic immune system distinguishes between commensal bacteria and pathogenic ones (and phages) in simpler two-component HPIs would also provide information that would help us to understand tripartite relationships with bacteriophages better.

#### Considering Spatial and Temporal Dynamics in Population Mixing

Spatial variability can be introduced by different tissues and microenvironments (Wikle and Royle, 2002). On a macroscopic level, this includes the separation of agents by anatomical barriers and the formation of densely packed bacterial biofilms (Leung and Weitz, 2017). This is therefore linked tightly to temporal dynamics and the presence of time delays. For example, time delays can occur as a result of the time taken for a pathogen or drug to cross anatomical barriers or changes in bacterial replication rates due to limited nutrient availability (e.g., phagosomal pathogens such as *Salmonella* will likely be more nutrient deprived than cytoplasmic or extracellular pathogens (Rienksma et al., 2019)). Leung et al. highlighted that it is population and concentration densities in the locality of infection that will directly impact on pathogenesis, disease prognosis and treatment efficacy, as opposed to overall loads per patient (Gog et al., 2012; Aljayyoussi et al., 2017; Leung and Weitz, 2017). Bacterial densities will also impact on the number of ‘contact events’ and bacteriophage infection and replication rates, as well as affecting pathogenesis and bacterial evasion of the immune system via quorum sensing signaling pathways and biofilm formation (Leung and Weitz, 2017). Biological systems are transient and dynamic and a snapshot image of a single time point therefore, will not be representative of the full time course of a treatment regimen.

Time delays are often not represented in *in vitro* liquid cultures, i.e., there are no anatomical barriers present, or the immune system is not fully represented. These spatial and temporal features have a tendency to be overlooked in modeling and when transitioning to *in vivo* studies therefore (Aljayyoussi et al., 2017). However, with so many outcomes dependent on population density, it would be greatly detrimental to the realism of a model should spatial and temporal features not be considered. For example, it was only the inclusion of temporal delays due to anatomical barriers in the tuberculosis models created by created by Aljayyoussi et al. (2017; discussed earlier) that produced treatment models with timescales that matched those seen in the real world. Therefore, we suggest that more rate-based experiments and models are required. For example, temporal resolution can be improved through the collection of live microscope data on infection rates at a single cell level (Gog et al., 2012) or collection of time course 16s RNA sequencing data (Wu and Ross, 2016) and the use of delay differential equations (DDEs; differential equations that will factor in a time delay; Hodyra-Stefaniak et al., 2015) in models. In particular, there is a need to better develop our understanding of bacteriophage transcytosis into the blood stream and around the rest of the body in order to make more accurate assumptions about dosages from local bacteriophage concentrations. To include these additional spatial features in a model, there are certainly occasions where a confluent lawn of eukaryotic cells or *in vitro* experiment will not suffice, as they do not represent anatomical barriers and the breadth of microenvironments within a eukaryotic host. In these situations, a more complex or animal model may be more beneficial (Keen and Dantas, 2018). If more complex or animal models cannot be used, great care should be taken to account for shortcomings when making conclusions and attempting to extrapolate data for use in an *in vivo* scenario.

#### Conclusion on Priority Areas for Future Phage Therapy Models

Collecting data on mutational heterogeneity and resistance to treatments, time delays and population densities can lead to large experimental and modeling demands, but it will also make models more representative of *in vivo* scenarios. The omission of any feature in a model should be an active decision in experimental design rather than it being just overlooked and the exclusion of key features from a model should be justifiable, based on the research question and the required complexity of the model. Omitted features should be handled carefully and accounted for as best as possible when extrapolating data and drawing conclusions.

## Discussion and Final Conclusion

Unlike antibiotics, there is a practically unlimited supply of novel bacteriophages. However, unpredictable *in vitro* and *in vivo* results currently hinder regulatory approval of phage therapies (Gordillo Altamirano and Barr, 2019). If mathematical modeling can be used alongside lab-based experiments to shortlist phage options, design experiments and enhance our confidence as to whether a phage therapy application would be successful and safe for *in vivo* models or humans prior to testing in the clinical environment, then this may smooth the road to widespread use.

Findings in the past few decades have highlighted more and more the complex relationship between not only a bacteriophage and bacterium, but also the bacteriophage and its eukaryotic host, with an appreciation of the synergy between the innate immune system and bacteriophages being key to understanding experimental outcomes (Tiwari et al., 2011; Górski et al., 2017; Roach et al., 2017; Van Belleghem et al., 2018). The success and realism of mathematical models and their ‘fit’ to real world data is something that has developed over time, with advances in experimental and computational technology, as well as our experimental capabilities and understanding (Bauer et al., 2009; Ewald et al., 2017) meaning our understanding of phage research is really only just reaching maturity (Lin et al., 2017). For example, advances in the collection of RNA and transcriptome data has transformed the way we develop FBA models and allowed the production of complex quantitative simulations of entire metabolic systems from more than one organism (Rienksma et al., 2018, 2019). Updates on pre-existing mathematical models are continuously being developed and published, factoring in larger data sets and new details on molecular-level host-pathogen interactions (Thiele et al., 2013; Rienksma et al., 2019). However, work remaining to be done includes the gathering of data on resistance and co-evolution (particularly related to mutational stochasticity and heterogeneity) and the spatial dynamics of population densities and biological time delays to aid further improvement of models. Although a number of models of phage therapy already exist, this additional data would open up the possibility of more realistic models, representing all three components in a clinical setting at once.

It is important to note that the data needed to create an informative model may already exist. For example, to create a DDE of a phage therapy scenario, data is needed on concentrations of susceptible and resistant bacteria, infected cells and free phage particles, rates of bacterial growth, phage infection, natural phage decay and bacterial resistance development at a given time, in addition to the length of phage latency periods and burst sizes, or these need to be fitted by the model (Cairns et al., 2009). Enough phage transcriptomic or genomic data to develop FBAs may also already exist (Kauffman et al., 2003). We therefore encourage collaboration between wet lab biologists and those well-informed in mathematical modeling in order to make the most of data that already exists and fill gaps where little more may be needed to complete a simulation. Looking forward, where biologists are aware of the types of quantitative or qualitative data needed to be input into a model, this might also help to more efficiently plan experiments to produce data that could be used by others *in silico*. The sharing of knowledge could lead to very fruitful synergism of resources and findings.

Mathematical models are of course not without their limitations, but collectively a plethora of information has been produced from modeling endeavors up to this date, including predicted treatment times and dosages (Aljayyoussi et al., 2017), a greater understanding of which scenarios will dominate in a biological setting (Gog et al., 2012; Tago and Meyer, 2016) and information on key agents which need to be present for a treatment to be successful (Roach et al., 2017) to give a few examples. There is hope therefore that mathematical modeling will play a hugely beneficial role in future research.

## Author Contributions

KMS: writing—original draft preparation. ATB and APS: writing—review and editing, supervision, project administration, and funding acquisition. All authors have read and agreed to the published version of the manuscript.

## Conflict of Interest

The authors declare that the research was conducted in the absence of any commercial or financial relationships that could be construed as a potential conflict of interest.

## Publisher’s Note

All claims expressed in this article are solely those of the authors and do not necessarily represent those of their affiliated organizations, or those of the publisher, the editors and the reviewers. Any product that may be evaluated in this article, or claim that may be made by its manufacturer, is not guaranteed or endorsed by the publisher.
